# Introducing variability in targeting the microtubules: Review of current mechanisms and future directions in colchicine therapy

**DOI:** 10.1002/prp2.616

**Published:** 2020-07-01

**Authors:** Esther Forkosh, Ariel Kenig, Yaron Ilan

**Affiliations:** ^1^ Department of Medicine Hebrew University‐Hadassah Medical Centre Jerusalem Israel

**Keywords:** colchicine, fibrosis, Inflammation, microtubules, variability

## Abstract

Microtubules (MTs) are highly dynamic polymers that constitute the cellular cytoskeleton and play a role in multiple cellular functions. Variability characterizes biological systems and is considered a part of the normal function of cells and organs. Variability contributes to cell plasticity and is a mechanism for overcoming errors in cellular level assembly and function, and potentially the whole organ level. Dynamic instability is a feature of biological variability that characterizes the function of MTs. The dynamic behavior of MTs constitutes the basis for multiple biological processes that contribute to cellular plasticity and the timing of cell signaling. Colchicine is a MT‐modifying drug that exerts anti‐inflammatory and anti‐cancer effects. This review discusses some of the functions of colchicine and presents a platform for introducing variability while targeting MTs in intestinal cells, the microbiome, the gut, and the systemic immune system. This platform can be used for implementing novel therapies, improving response to chronic MT‐based therapies, overcoming drug resistance, exerting gut‐based systemic immune responses, and generating patient‐tailored dynamic therapeutic regimens.

**Abbreviations**
2‐ME: 2‐methoxyestradiol
ACF7: (also termed MACF1) microtubule‐actin crosslinking factor 1
APC: adenomatosis polyposis coli
APCs: antigen presenting cells
CA: combretastatin A
CBSI: colchicine binding site inhibitors
CGRP: calcitonin gene‐related peptide
CORE: colchicine fore recurrent pericarditis
CORP: colchicine for recurrent pericarditis
CPPD: calcium pyrophosphate dehydrate
CTGF: connective tissue growth factor
EB1: end binding protein 1
FMF: familial Mediterranean fever
GDP: guanosine diphosphate
GTP: guanosine triphosphate
IL: interleukin
IS: immune synapse
MAP: microtubules associated proteins
MSU: monosodium urate
MT: microtubules
MTOC: microtubules‐organizing center
NF‐kB: nuclear factor kappa‐light‐chain‐enhancer of activated B cells
NLRP3: nod‐like receptor protein 3
PPS: post pericardiotomy syndrome
PTM: post‐translational modifications
RCT: randomized clinical trial
SCFA: short chain fatty acids
SxIP: Xer‐x‐Ile‐Pro
TCR: T‐cell receptor
TGFβ: tumor growth factor β
TIP: microtubular plus end tracking protein
TNFα: tumor necrosis factor α

## 1 INTRODUCTION

Microtubules (MTs) are highly dynamic cytoplasmatic polymers that constitute the cellular cytoskeleton and the internal structure of cilia and flagella.^1^ MTs are composed of αβ‐tubulin heterodimers and exhibit diverse structural and functional properties in different cell types.^2^ They are involved in mitosis and meiosis as constituents of the mitotic spindle and in cellular transport systems.^3^ In polarized interphase cells, MTs are disproportionally oriented from the MT‐organizing center (MTOC) toward the site of polarity.^4^ The dynamic behavior of MTs constitutes the basis for multiple functions, including cellular motility, cytoplasmic transport, and cell division.^5^ MT dynamics are altered in cancer cell divisions and linked to chromosomal instability, aneuploidy, and development of drug resistances. Dynamic instability is a feature of variability that characterizes the function of MTs.^5^

Colchicine is a neutral lipophilic alkaloid compound found in a variety of plants. It is most abundant in *Colchicum autumnale*, a poisonous plant in the lily family.^6^ *C autumnale* was first documented in 1500 BCE in the Egyptian medical manuscript *Ebers papyrus* as a treatment for joint pain and swelling. Colchicine is contained in the seeds, flowers, and corms of *C autumnale*. In 1820, the active ingredient was isolated.^7^ Its structure was determined by X‐ray crystallography and tubulin was identified as the target for its activity.^8^, ^9^ The composition of the amino acids heterodimer was determined using MTs extracts.^10^ Here, we review some of the functions of colchicine and its current medical use and present a platform for introducing variability by using it to target MTs, thereby improving its therapeutic effects.

## 2 MICROTUBULES AS A TARGET FOR THERAPEUTIC INTERVENTION

MTs were proposed as therapeutic targets primarily for anti‐inflammatory and anti‐cancer properties. Targeting the MTs and the subsequent disruption of normal function of the mitotic spindle promotes an anti‐cancer effect. MT‐interfering drugs have been shown to be effective chemotherapeutics.^5^ Anti‐cancer drugs suppress the dynamics of the mitotic spindle, leading to mitotic arrest and cell death. Minor alteration of MT dynamics can engage the spindle checkpoint, thus arresting cell‐cycle progression at mitosis and subsequently leading to cell death.^11^ Altered MT stability, expression of diverse tubulin isotypes, and altered post‐translational modifications have been described in various cancers, and they may correlate with poor prognosis and chemotherapy resistance.^12^ The diversity of tubulin structure is manifested in tubulin isotypes and post‐translational modifications (PTMs). The altered expression of these isotypes and PTMs are associated with the development of resistance toward anti‐malignant medications.^2^

MTs are also involved in the function of both the innate and adaptive immune systems, making them potential targets for immunomodulation.^13^ MTs, through crosstalk with the antigen‐receptor signal transduction machinery, are associated with the formation of the immune synapse (IS).^14^, ^15^ The IS is formed by the polarization of MTs following T‐cell receptor (TCR) activation. Upon recognition of a peptide‐MHC complex by antigen presenting cells(APCs), TCR microclusters move along MTs to the IS center.^16^ The IS regulates T cell activation by interacting with APCs.^17^, ^18^ While immune cells lack cilia, the IS formation utilizes machinery associated with ciliogenesis, including the nucleation of MTs at the centrosome.^19^ MTs are essential for directing cytokine to the IS, and the formation of MTs is reciprocally induced by cytokine stimulation.^20^ The IS, contrary with the stable cilia, exists only for a few minutes but has the ability to rapidly polarize MTs, enabling fast secretion of cytotoxic granules in cytotoxic T cells.^21^ In addition, MTs are associated with cellular migration, an essential function of neutrophils and other immune cells.^22^ MTs also play a pivotal role in the formation of the inflammasome, a pro‐inflammatory multiprotein.^23^ Immature CD4 + CD8+ thymocytes fail to polarize their MTOC due to the inhibition of MT stability by glycogen synthase kinase 3.^24^ The mechanosensing ability of B cells is also dependent upon MTs, as they are required for the trafficking of B cell receptor‐antigen complexes.^25^, ^26^ MTs are also essential for organizing natural killer cell receptors, establishing cellular polarity, coordinating immune receptors with integrin‐mediated signaling, and directing secretion of lytic granules and cytokines.^27^

Taken together, the data show that MTs have multiple roles in a wide variety of cellular functions.

## 3 TARGETING OF MICROTUBULES BY COLCHICINE

Colchicine has anti‐inflammatory, anti‐mitotic, and anti‐fibrotic activity, however, its narrow therapeutic index and the overlap between therapeutic and toxic doses has prohibited its widespread use.^28^ Colchicine is absorbed rapidly through the gastrointestinal tract and is eliminated primarily by hepatic metabolism, followed by biliary excretion with enterohepatic re‐circulation. In addition, 5%‐20% of the drug is excreted by the kidneys. It is effective in doses of 0.015 mg/kg but toxic in doses greater than 0.1 mg/kg, which may cause gastrointestinal manifestation, coagulation disorders, and bone marrow aplasia.^29^ Doses exceeding 0.8 mg/kg are lethal.^30^

The primary therapeutic mechanism of colchicine is tubulin disruption.^31^ Colchicine binds at the interface of soluble α and β tubulin subunits and inhibits further polymerization by adding to the end of MTs and arresting MT growth in low doses, and by promoting MT degradation in higher doses.^28^, ^32^ Colchicine blocks mitosis, diverting the cells to undergo an abnormal cell cycle, which terminates in nuclear envelope breakdown and undivided centromeres. Cells in different stages of mitosis exhibit different sensitivities to colchicine. While in lower concentrations, prophase cells are more sensitive, and at higher concentrations, colchicine blocks all cells in metaphase.^28^, ^32^, ^33^

## 4 COLCHICINE EXERTS AN ANTI‐INFLAMMATORY EFFECT

By inhibiting MT arrangement, colchicine disrupts the fundamental functions of immune cells. It blocks neutrophil adhesion, therefore blocking neutrophil migration and recruitment, by altering the distribution of E‐selectin and the levels of L‐selectin.^34^ Colchicine also reduces neutrophil deformability by reducing cytoplasmatic MT levels. This alters the ability of neutrophils to migrate through small pores, which is crucial for extravasation in response to inflammatory signals.^35^ Colchicine can antagonize intracellular MT‐mediated trafficking.^36^

MT stability is associated with the nuclear factor kappa light chain enhancer of activated B cells (NF‐kB), the activation of which is induced by tumor‐necrosis factor α (TNF‐α). An association between MT stability and the regulation of NF‐κB signaling has been documented. Increased MT stability following paclitaxel administration resulted in NF‐kB induction, even in the absence of TNF‐α. However, paclitaxel and TNF‐α induction of NF‐kB is reduced by colchicine,^37^ as colchicine suppresses TNF‐α receptors on macrophage surfaces.^38^ Cells treated with colchicine show a reduced capacity for antigen transport to the trans‐Golgi area compared to cells treated with the MT polymerizing agent, paclitaxel.^39^

Gout is a common form of inflammatory arthritis, effecting 3%‐6% of men and 1%‐2% of women in western developed countries. It is commonly associated with hypertension, diabetes, and obesity^40^ and is characterized by an interleukin (IL)‐1β‐mediated inflammatory reaction to MSU crystals, resulting in acute, and later chronic, arthritis.^40^, ^41^ Low dose colchicine is effective in treating acute flairs of gout arthritis. Complete response (defined as 50% pain relief without the need of other medications) has been recorded in 37.8%, compared to 15.5% in the placebo group.^42^ Furthermore, colchicine prophylaxis for at least six months while initiating urate‐lowering treatments has been shown to reduce the risk of acute attacks in two randomized clinical trials (RCTs).^43^ Similarly, when colchicine is administered in acute attacks of calcium pyrophosphate dihydrate (CPPD) disease, there is some evidence supporting its preventive role in recurrent cases.^44^, ^45^ Three small RCTs showed pain and function improvement in osteoarthritis patients treated with colchicine.^32^ Significant improvement in visual analog scale and western Ontario and McMaster university scores in patients treated with 0.5 mg colchicine twice daily was recorded.^46^ A recent RCT involving 109 patients with osteoarthritis showed that colchicine reduced biomarkers of inflammation and high bone turnover disease, known to be associated with osteoarthritis progression. However, it did not result in clinical improvement in 16 weeks period when tested.^47^

A Monosodium urate (MSU) and CPPD crystal activate the production of IL‐1β in the nod‐like receptor protein‐3 (NLRP3) inflammasome, resulting in gout and pseudo‐gout attacks.^41^ Colchicine disrupts the NLRP3 inflammasome activation by inhibiting the transport of mitochondria to the endoplasmic reticulum, thus preventing co‐localization of NLRP3 and an apoptosis‐associated speck‐like protein containing a caspase recruitment domain, which is necessary for the production of mature IL‐1β.^23^ Colchicine also blocks the production of IL‐1β by caspase‐1 activation by inhibiting the RhoA/Rho effector kinase pathway through cytoskeleton rearrangement.^32^ Additionally, colchicine selectively suppresses MSU‐induced superoxide production by neutrophils.^48^

Colchicine is effective in the treatment of auto‐inflammatory diseases. Familial Mediterranean fever (FMF), the most common auto‐inflammatory disease, is an autosomal recessive disease caused by a mutated MEFV gene. The MEFV gene encodes the protein pyrin, an important immunoregulatory component of the inflammasome.^49^ Colchicine is a first line of treatment for FM patients, because it reduces the recurrence rate of FMF flares.^50^ In addition, it prevents the development of AA amyloidosis, a dreadful sequalae of FMF.^51^ More recently, its use was extended to the treatment of periodic fever with aphthous stomatitis, pharyngitis, and adenitis^52^ and is also used in the treatment of mucocutaneous and articular manifestations of Bechet's disease.^32^, ^52^

Colchicine prevents pericarditis recurrence and may have a role in the prevention of post pericardiotomy syndrome (PPS). When added to the conventional treatment in 120 patients experiencing their first pericarditis episode, colchicine significantly reduced the recurrence rate.^53^ Administration of colchicine following the first recurrence of pericarditis was tested in the CORE and CORP trials and was found to reduce symptom persistence and recurrence rates.^53^, ^54^ Similar results were achieved in patients with multiple recurrences in the CORP‐2 trial.^55^ The administration of colchicine prior to cardiac surgery has also been found to reduce PPS.^56^, ^57^

Inflammation plays a role in atherosclerosis and its complications. In a randomized, double‐blind trial, 4745 patients enrolled within 30 days after myocardial infarction were followed for a median of 22.6 months. Colchicine at a dose of 0.5 mg daily lowered the risk of ischemic cardiovascular events.^58^ The primary end point, death from cardiovascular causes, resuscitated cardiac arrest, myocardial infarction, stroke, or urgent hospitalization for angina leading to coronary revascularization, occurred in 5.5% of the patients in the colchicine group, as compared to 7.1% of the placebo group. Diarrhea was reported in 9.7% of the patients in the colchicine group and 8.9% of the placebo group, and pneumonia was reported in 0.9% of the colchicine group and in 0.4% of those in the placebo group.^58^

## 5 THE ROLE AND MECHANISM OF ACTION OF COLCHICINE IN CANCER TREATMENT

Cancer cells are characterized by a high mitotic rate, making them susceptible to MT‐targeting agents. There are three general classes of drugs which bind to the tubulin subunit and block the cell cycle: depolymerizing agents like colchicine, drugs that induce alternate polymers like vinca alkaloids, and drugs that stabilize MTs like taxol.^59^, ^60^, ^61^ The consequences of disrupting MT dynamics are similar for all three types and include metaphase arrest and induction of apoptosis.^62^ Colchicine interacts with tubulin to disrupt the formation of tumor vasculature and damage pre‐existing blood vessels within the tumor.^63^ Colchicine also reduces mitochondrial metabolism in cancer cells through the inhibition of the voltage‐dependent anion channels of the mitochondrial membrane.^64^, ^65^ Large numbers of compounds that interact with the colchicine binding site were reported to be effective in killing cancer cells, including Indanocin, which disrupts the mitotic spindle, arresting cells in early prophase.^62^, ^66^

Estrogen metabolite 2‐methoxyestradiol (2‐ME) and the oxadiazoline derivative A204197 arrest the division of cells. 2‐ME is a potent inhibitor of tumor vasculature and tumor cell growth. Chemical modification at the 3 and 17 positions of 2‐ME generate the metabolically stable analog ENMD‐1198, which binds to the colchicine binding site in tubulin, inducing G2/M cell cycle arrest and apoptosis and reduces hypoxia‐inducible factor‐1α levels.^67^ Several natural products that bind the colchicine domain and disrupt MTs are under investigation, including combretastatin‐A‐4 3‐O‐phosphate (CA‐4‐P), CA‐1‐P, ZD6126 and AVE8062A.^11^, ^30^

Colchicine shows significant anti‐proliferative effects in hepatocellular carcinoma cell lines and on fibroblasts in the tumor microenvironment. These effects are associated with the up‐regulation of anti‐proliferative genes, including the tumor suppressor AKAP12 and the tumor growth factor β2 (TGFβ2), which suppresses cell cycle progression at the G1 phase and MX1,promoting cell death.^64^ Colchicine also upregulates the DUSP1 gene, inhibiting the cellular proliferation and inducing apoptosis in gastric cancer cells.^68^

## 6 THE ANTI‐FIBROTIC EFFECTS OF COLCHICINE

In addition to its anti‐inflammatory properties, colchicine exhibits anti‐fibrotic effects in various organs, including the heart, kidneys, lungs, and liver. In one study, myocardial infarction size and myocardial fibrosis were decreased in myocardial ischemia murine model after colchicine administration.^69^ Colchicine administration in ST segment elevation myocardial infarction resulted in smaller infarct size and lowered levels of myocardial biomarkers.^70^ However, it did not attenuate fibrosis and left ventricular remodeling in spontaneous hypertensive rats, which are known to suffer from increased fibrosis.^71^ Colchicine decreased fibrosis and enhanced activity of matrix metalloproteinase‐2 in hamsters with experimental Chagas disease.^72^

Colchicine administration in the unilateral ureteral obstruction model results in suppressed interstitial fibrosis, decreased fibrogenic gene expression, and reduction in the levels of caspase‐3, fibronectin, and ED‐1.^73^, ^74^ The upregulation of profibrotic cytokine connective tissue growth factor (CTGF) by mechanical strain is dependent on RhoA activation. Colchicine inhibits RhoA activation, resulting in attenuated glomerular fibrosis and sclerosis with decrease in Type 1 collagen, fibronectin, and CTGF levels.^75^ Moderate anti‐fibrotic effect has been shown in anti‐glomerular basement membrane disease model in rabbits.^76^ The levels and effect of the pro‐fibrotic TGF‐β are decreased in colchicine‐treated animal models.^75^, ^77^

Studies of colchicine in bleomycin‐treated rats showed contradicting results regarding lung fibrosis improvement.^78^, ^79^ Colchicine treatment in patients with idiopathic pulmonary fibrosis has not been shown to be beneficial.^80^ In a randomized prospective trail, colchicine showed a trend for improved survival in these patients, albeit not significant, when compared with high‐dose prednisone. A combination of colchicine with D‐penicillamine and prednisone did not result in clinical improvement.^81^

Colchicine improved hepatic fibrosis in rat models through the inactivation of stellate cells and inhibition of TGF‐β expression. A systemic review summarizing 15 RCTs concerning colchicine administration in alcoholic and nonalcoholic liver fibrosis showed a lack of beneficial effect on liver histology, mortality, or rate of complications.^82^

## 7 RESISTANCE TO COLCHICINE THERAPY

Despite regular use, 10%‐30% of patients with FMF do not respond to colchicine, even when used at the highest tolerable dose.^83^, ^84^, ^85^, ^86^, ^87^ There is no standard, validated definition for colchicine resistance, and proper definitions for attack‐free periods and persistence of high acute phase reactants are still lacking.^85^ Colchicine does not exert an immediate effect when administered during an acute attack of FMF, suggesting that higher concentrations and prolonged treatment periods may be required for to achieve an effect on neutrophil function and for a resulting therapeutic effect.

Several mechanisms have been proposed as the primary and secondary lack of response to colchicine. Resistance to colchicine is mostly observed in patients with specificMEFV genotypes.^88^ Nonresponsiveness is associated with genetic heterogeneity in single nucleotide polymorphisms (SNPs) of ABCB1.^84^ In patients with C genotype, 3435C to T polymorphism is more resistant to colchicine than patients with the TT allele.^86^ Certain pyrin mutations prevent colchicine from binding to 14‐3‐3 protein relieving the inhibition of the pyrin inflammasome.^89^ SNP studies of the binding site of colchicine on beta tubulin suggest that substitutions of two amino acids, namely A248T and M257V, reduce the binding energy of colchicine twofold.^90^

Vitamin D deficiency in FMFwas proposed to contribute to the lack of response to colchicine.^87^ Blood group A had 1.5‐fold higher incidence of FMF and a better response to colchicine treatment than the non‐A blood group, while patients in the O blood group prominently exhibited colchicine resistance.^91^

Cellular transport of colchicine is mediated by the P‐glycoprotein (P‐gp) efflux pump encoded by the multiple drug resistance‐1 gene.^92^ P‐gp is a membrane‐associated ATP‐binding cassette transporter that is overexpressed in tumor cell lines, including tissues of the liver, kidney, and gastrointestinal tract. Tumor resistance to colchicine occurs via its P‐gp induction activity, leading to an active efflux of colchicine from tumor cells, which causes a decrease in its cytotoxicity. Mutations in tubulin and overexpression of βIII‐tubulin isoform may underlie resistance.^67^, ^84^ Targeting the colchicine binding site inhibitors (CBSIs) with anticancer compounds, have the ability to overcome both P‐gp and β‐III tubulin‐mediated resistance and may overcome drug resistant tumors.^67^ MCF cell lines that are exposed to gradual increments of colchicine treatment induce a colchicine‐resistant response. In comparison to the parent MCF‐7 cells, the dynamic instability of MTs are suppressed, and β‐tubulin isotypes are associated with reduction in colchicine binding.^93^ The chemical compounds AS1712 and RJ‐LC‐15‐8 are CBSIs that can overcome the P‐gp efflux pump and β‐tubulin alterations.^94^

Overall, the data exemplify that the problem of colchicine resistance in a significant number of the patients is due to adverse suite of mechanisms.

## 8 DYNAMIC INSTABILITY IS A FEATURE OF BIOLOGICAL VARIABILITY, WHICH CHARACTERIZES THE FUNCTION OF MICROTUBULES

MTs are typically comprised of 13 protofilaments assembled from αβ tubulin heterodimers that stack head to tail. The protofilaments associate in parallel, giving rise to a polar cylinder.^95^ They are characterized by their special dynamic nature which allows the cell to rapidly reorganize the cytoskeleton when necessary. Mitchison and Kirschner observed that at any point in time, stochastic switching between growing and shrinking states occurs, behavior known as dynamic instability.^96^, ^97^, ^98^ α‐ and β‐tubulins each contain a binding site for the energy carrier guanosine triphosphate (GTP) at the longitudinal interface between the subunits. GTP is the driving force for polymerization. For MTs to grow, the cell must consume energy to keep the concentration of GTP tubulin high. α‐tubulin binds the GTP at the nonexchangeable (N)‐site, which is located in the intra‐dimer interface, where it plays a structural role. β‐tubulin binds the GTP at the exchangeable (E)‐site, which is exposed in the unassembled dimer.^99^ After subunits are incorporated into MTs, the GTP of the β‐tubulin subunit is hydrolyzed, and phosphate is released, which converts the GTP tubulin into a guanosine diphosphate (GDP)tubulin. The hydrolysis of GTP lags behind the binding of new GTP tubulins, creating a cap of GTP tubulin at the MT end, termed the plus end.^100^, ^101^ A cap of GTP tubulin at the E‐site can stabilize the plus end of MT structure and promote growth, while its disappearance by GTP hydrolysis makes MT lattices unstable and prone to depolymerization.^99^ Diverse conformational states distinguish the two phases from one another. When MTs grow, the exchange of GTP into its active site straightens the dimer, facilitating its incorporation into a sheet at the growing end of the MT.^102^ When MTs shrink, the GTP cap is lost, and the GDP tubulin relaxes into a curved conformation (12° bend per αβ‐tubulin) that does not fit into the straight wall of the MT and falls off the polymer rapidly. The swiftness with which the GDP‐MT structure collapses defines the phenomenon known as catastrophe.^100^

Rapid remodeling of the MT cytoskeleton is regulated by MT‐associated proteins (MAPs), whose functions are to add and remove tubulin subunits.^103^, ^104^ Mitotic centromere‐associated kinesins are ATPase proteins that target the MT protofilament end rapidly and catalytically depolymerize MTs by accelerating the rate of dissociation by 100‐fold and removing the GTPcap.^102^, ^105^ Proteins with an opposite action, such as XMAP215, act as polymerases and can increase the association rate up to tenfold.^102^ XMAP215 is a long, thin molecule that forms small, curved protofilaments by binding multiple tubulins to the growing end.^106^

The growth and shortening rates of MTs are highly variable. The mean growth rate of individual MTs increases with increasing tubulin concentration. The variability is too large to be attributed to known random measurement error; hence, it must be an inherent character of the MTs.^107^, ^108^ This randomness was proposed to be required for cell plasticity and normal cell function and organization. The variability of growth and shortening rates was proposed to result from structural changes that are transient in time and associated with the dynamics of the growing and shortening ends. Variability is apparently unaffected by imperfections in the lattice, as it does not become more common at higher growth rates. Defects incorporated into the lattice are removed rapidly enough to keep up with the increased rate of subunit addition^108^, ^109^, ^110^

## 9 MICROTUBULES CONTRIBUTE TO PRECISION AND PLASTICITY IN CELLULAR TRANSCRIPTION

MTs and MAPs underlie some of the mechanisms associated with cellular plasticity via their roles in cellular proliferation, structural changes, and molecule trafficking. Cellular identity is a fundamental feature of biology; however, cellular plasticity in the context of tissue injury is a well‐recognized phenomenon. Plasticity is a complex process that differs between tissue types. Mature cells retain the potential to undergo lineage reversion or trans‐differentiation, which enables them to convert into cells of a more distant lineage.^111^ The multilineage potential of epithelial stem cells changes depending on whether it exists in its resident niche and responds to normal tissue homeostasis or is mobilized to repair a wound.^112^ Cells of the nervous system can modify their structure and functionality following injury and in response to different triggers, a feature of learning and development.^113^ In several cell types, plasticity is inhibited by complex genetic mechanisms after the cell is fully developed. In the nematode *C elegans*, a neuronal identity‐inducing transcription factor, CHE‐1, cannot activate target genes in mature cells due to multiple prohibitory proteins, including ubiquitin hydrolase usp‐48, the chromatin‐related factor H3K79, MAPK‐type protein kinases, and nuclear localized O‐GlcNAc transferase.^114^ In *Drosophila*, the broadly expressed Hox transcription factor Ubx binds and regulates genes in a tissue‐specific manner.^115^

During cell division, kinetochores attach to the plus ends of MTs. The spindle assembly checkpoint ensures that chromatid separation is activated after all chromosomes are attached to spindle MTs. As long as the chromosomes are not bi‐oriented, there is no tension, and the kinetochore‐MT attachment is close to the centromeric pool of protein kinase Aurora B. This enables phosphorylation of key regulatory proteins at the kinetochore by Aurora B, resulting in detachment of associated MTs. The MT‐kinetochore attachment is unstable during prometaphase and switches to a more stable connection in metaphase, due to the Cyclin‐A degradation timer that starts at prometaphase and ends at metaphase.^116^ Checkpoint proteins bind to both the kinetochore and the MT end to ensure precise chromosome division. It involves binding of Bub3 to the Spc7 MELT array. Once the occupancy of Spc7 (KNL1) by Bub3 drops, the levels of potent anaphase promoting complex inhibitor, the mitotic checkpoint complex, decrease so that anaphase can progress.^117^

MT plus end tracking proteins (+TIPs) are localized to the MT plus end in an end‐binding protein‐1 (EB1)‐dependent manner using a short polypeptide motif, Ser‐x‐Ile‐Pro (SxIP).^118^ The attachment of EB1 to TIP150 promotes the stability of MT plus ends in mitosis. This interaction is controlled by the p300/CBP‐associated factor acetylation, which is a timely process. Persistent acetylation perturbs the EB1‐TIP150 interaction and accurate metaphase alignment.^119^ Dynamic acetylation of the highly conserved C‐terminal, K220, of EB1 regulates the binding of EB1 to various TIPs containing SxIP motif during mitosis, including TIP150. This promotes accurate kinetochore‐MT interaction.^120^ The data show that untimely persistence of EB1 acetylation delays metaphase alignment and results in mitotic arrest. In syncytial cells, MTs help to compartmentalize specific cell territories for the timing of nonsynchronized nucleus division.^121^

Neuronal plasticity requires that signals generated in the synapse are transferred to the nucleus. Synaptic activation triggers NF‐kB retrograde transport by the dynein/dynactin motor complex. This process is blocked by overexpression of dynamitin, which dislocates dynein from MTs. MT‐disrupting drugs inhibit this process and prevent NF‐kB‐dependent transcription activity in the nucleus.^122^ Location‐dependent effects of transcription factors are assisted by neuronal MTs. For example, ELK1 is a pro‐apoptotic factor within the cytoplasm and a pro‐differentiation factor in the nucleus that directly binds with tubulin.^123^, ^124^

The data support the notion that MTs contribute to the underlying mechanisms of cellular plasticity.

## 10 MICROTUBULES IN THE GUT AS THERAPEUTIC TARGETS

The gut provides potential targets for generating anti‐inflammatory signals without the burden of immunosuppression and systemic adverse reactions, which limit the use of current anti‐inflammatory drugs.^125^, ^126^, ^127^ MTs play an important role in the structure and function of the gut. This combined with their crucial functions in the immune system, allows gut MTs to be prominent novel targets for anti‐inflammatory treatments.^13^, ^128^ Gut MT‐directed therapy can potentially provide a systemic effect without being absorbed through the local intestinal cells and the local immune system.

MT rearrangement plays a part in gut elongation during development and is triggered by Jun N‐terminal kinase signaling.^129^ The cytoskeleton provides lasting support for gut structure and function. Gut epithelial cells are polarized and have distinct apical and basolateral domains. MTs in the polarized epithelial cells display an apical‐basal orientation with the minus end anchored in the apical domain.^130^ This structure is crucial for apical membrane protein trafficking.^131^ MT‐actin crosslinking factor 1(MACF1 or ACF7) regulates cytoskeletal focal adhesion and migration. ACF7 knock‐out increases gut permeability and epithelial cell apoptosis in mice models. In addition, its absence increases the gut inflammatory response to a high‐fat diet.^132^ Abnormal gutMT function and structure are noted in cells with adenomatous polyposis coli (APC) mutation, which is prevalent in sporadic colorectal tumors. Abnormal APC reduces MT stability and particularly decreases modified MTs in the migratory edge of the cell periphery. This results in reduced cellular migration and cellular protrusion.^133^

The intestine constitutes the largest interface between the human body and the surrounding world. Gut barrier disruption contributes to the pathogenesis of a variety of inflammatory gastrointestinal disorders, such as inflammatory bowel disease, celiac disease, and food allergies.^134^ Intestinal barrier dysfunction contributes to the development of multiorgan failure in septic patients.^135^ Oxidants induce disruption of epithelial‐barrier integrity by disrupting the cytoskeleton through the activation of the lambda isoform of protein kinase C. Epithelial growth factor protects from oxidant disruption of the gut barrier.^136^

The interplay between bacteria and MTs is of relevance both to the understanding of the microbiota in health and during infections. Short chain fatty acids (SCFAs) are produced by the gut microbiota and dysregulate the balance of β‐tubulin isotypes toward those associated with MT depolymerization.^137^ *Clostridium difficile* toxins induce both tubulin deacetylation and MT disassembly, promoting barrier dysfunction and microtubular rearmament and resulting in membrane protrusion, which increases its adhesion.^138^, ^139^Colchicine administration prevents *Escherichia coli* cellular internalization and translocation.^140^ *Klebsiella pneumonia* invades through a transcellular mechanism that is dependent on actin and MTs and is inhibited by nocodazole and by Rho inhibitors.^141^ *Campylobacter jejuni*, the leading cause of food borne illness, utilizes an actin‐independent microtubular mechanism for cell invasion.^142^ Absence of APF7, a cytoskeletal cross‐linking factor, results in increased LPS levels in blood.^132^

MTs are also involved in the enteric nervous system, which is tightly linked to the function of epithelial cells and immune cell in the intestine. The transport of mitochondria inside enteric neurons in Guinea pigs has been shown to be blocked by colchicine‐induced MT disruption.^48^ Calcitonin gene‐related peptide (CGRP) is a neurotransmitter in the enteric sensory system and is elevated in food allergy models. CGRP enhanced mucosal MT reorganization which in turn augmented IgE‐independent mucosal mast cell degranulation, contributing to food allergy development.^143^

## 11 INTRODUCING VARIABILITY IN TARGETING MICROTUBULES FOR IMPROVING THE EFFECT OF COLCHICINE

Variability is inherent to biological systems and is considered a part of the normal function of cells and organs.^144^, ^145^, ^146^ The dynamic instability of MTs exemplifies the variability in their structure and function.^147^ Variability contributes to cell plasticity and is a method used for overcoming errors in the assembly and tasks at the cellular and potentially whole organ levels.^145^, ^146^, ^148^, ^149^, ^150^ Variability also characterizes the response to certain therapies.^149^ The introduction of variability was proposed as a method for overcoming partial or complete loss of effect to medications for chronic conditions. This phenomenon may partially be related to chronotherapeutic effects.^151^, ^152^, ^153^ Whether chronobiology is linked to MT behavior is yet to be determined.^154^ Ongoing clinical trials (NCT03843697; NCT03747705) are evaluating effects of introducing variability in patients with inflammatory bowel disease who lost their response to anti‐TNFs, and in patients with epilepsy who lost response to anti‐epileptics. Data from these studies is expected to show an ability to improve response to medications by applying variability‐based therapeutic regimens.

**FIGURE 1:**
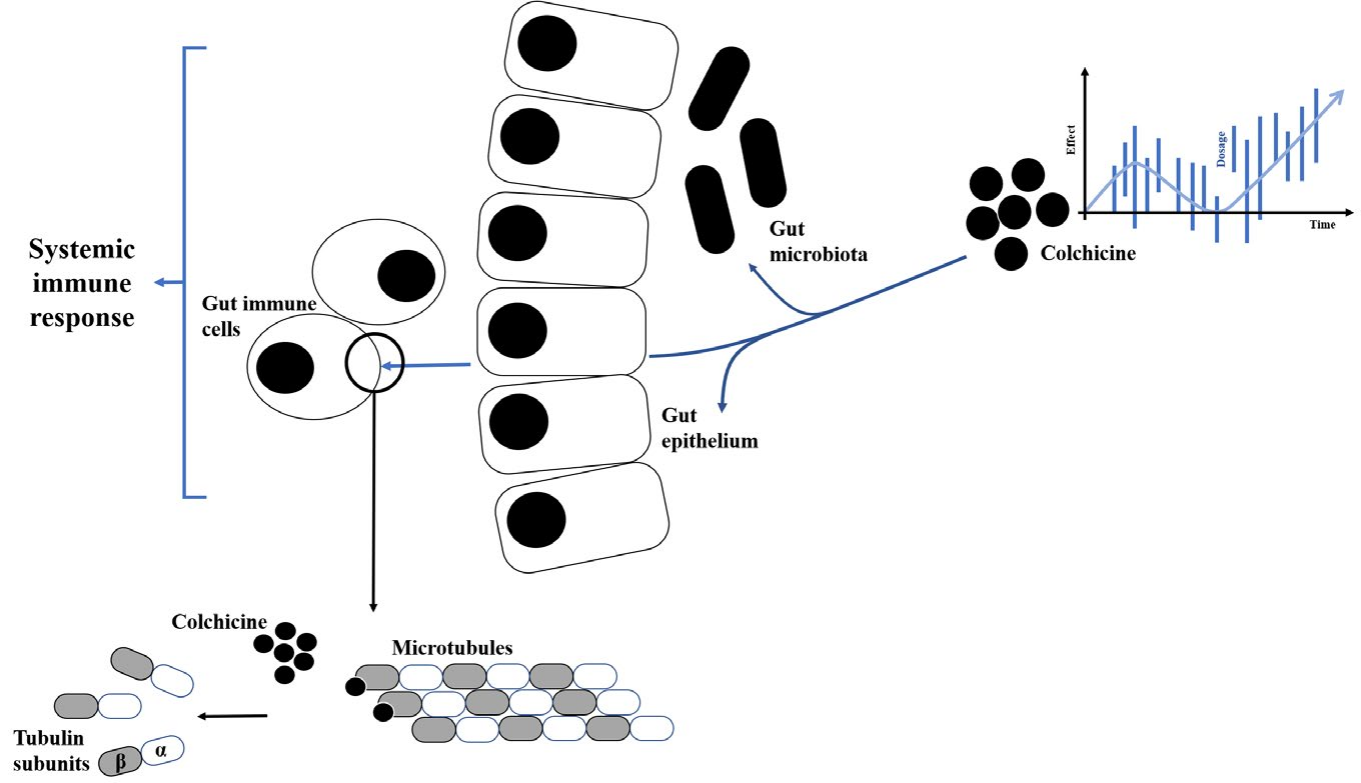
A schematic presentation of a platform comprising MTs in cells of the gut wall, the microbiome, and the intestine and systemic immune systems. Introducing variability in the dosing and intervals of administration of low nonabsorbable dose of colchicine is presented as a method for targeting this platform. Quantifying cellular and whole‐organs variability patterns as well as chronobiology‐based signatures are being implemented into the drug regimens

Figure 1 presents a schematic presentation of a platform comprising MTs in cells of the gut wall, the microbiome, and the intestine and systemic immune systems. Introducing variability in the dosing and intervals of administration of low nonabsorbable dose of colchicine is proposed as a method for targeting this platform. The suggested platform can be designed based on quantifying cellular and whole‐organs variability patterns as well as chronobiology‐based signatures in a personalized way. It is expected that the incorporation of these platforms into the therapeutic interventions, may support a long‐term sustainable response to chronic MTs‐based therapies, overcome drug resistance, and generate patient‐tailored dynamic therapeutic regimens.

In summary, the data on the importance of MTs in various cellular functions, along with the dynamic instability characterizing their structure and function make them attractive targets for the introduction of novel therapeutic platforms. Improving the response to colchicine and other MTs‐targeting drugs may take advantage of these platforms for improving the anti‐inflammatory and anti‐malignant effects of these drugs.

## DISCLOSURE

YI is the founder of Oberon Sciences and is a consultant for Teva, ENZO, Protalix, Betalin Therapeutics, Immuron, SciM, Natural Shield, Tiziana Pharma, Plantylight, and Exalenz Bioscience.

## AUTHORS CONTRIBUTION

YI AK and SZ anlayzed the source data and prepared the manuscript.
